# The Impact of Young Entrepreneurs’ Network Entrepreneurship Education and Management System Innovation on Students’ Entrepreneurial Psychology

**DOI:** 10.3389/fpsyg.2021.731317

**Published:** 2021-11-10

**Authors:** Zeyu Wang, Run Tang, Xin Cheng

**Affiliations:** ^1^Institute of Quality Development Strategy, Macro-Quality Management Collaborative Innovation Center in Hubei Province, Wuhan University, Wuhan, China; ^2^School of Music and Dance, Yantai University, Yantai, China; ^3^School of Public Administration, Zhongnan University of Economics and Law, Wuhan, China

**Keywords:** entrepreneurship education, entrepreneurship management system, teaching methods, students’ entrepreneurship psychology, cognition of entrepreneurs’ professional status

## Abstract

The purpose of the study is to solve the problems existing in entrepreneurship education and management under computer technology. The teaching content of entrepreneurship education in colleges and universities is proposed. Since entrepreneurship education is practical, the auxiliary mechanism of entrepreneurship education also needs to be highly integrated with entrepreneurship practice. First, the network entrepreneurship teaching and management system is constructed, and students’ entrepreneurial creativity, communication ability, leadership ability, and qualities are taken as the research object. Second, the traditional teaching method, case study method, and scene simulation method are used to analyze and discuss the influence of the entrepreneurial teaching mode, entrepreneurial experience, and entrepreneurial ability on students’ entrepreneurial psychology. Finally, the questionnaire survey is used to conduct the relative sample *t*-test (Student’s *t*-test), and the influence of three teaching methods on students’ learning effects is analyzed. The influence of the three teaching methods on students’ entrepreneurial psychological states is further analyzed by the statistical method. The experimental results show that the test result of the scene simulation method and the traditional teaching method is 0.584, the test result of the scene simulation method and the case study method is 0.842, and the test result of the case study method and the traditional teaching method is 0.595. This shows that the scene simulation method has a significant impact on students’ entrepreneurial psychology and their entrepreneurial ability. In addition, students’ cognition of professional status significantly affects their entrepreneurial psychology and attitudes, and the correlation coefficient is 0.576. Therefore, it is suggested that colleges and universities should adopt the scene simulation method to improve the teaching quality of entrepreneurship education and strengthen students’ cognition of professional status and their entrepreneurial practice.

## Introduction

Innovation and entrepreneurship education in colleges and universities is opened to cultivate students’ entrepreneurial consciousness, innovative spirit, and entrepreneurial ability, which meets the requirements of society. It is designed for the groups who intend to start a business, have started a business recently, and have successfully started a business (Ratten and Usmanij, 2021), so the course should be carried out hierarchically to cultivate the talents differently with the basic entrepreneurial literacy and creative personalities (Jena, 2020). With the transformation and development of the social economy, the new economic model plays an important role in promoting social development. As a generation growing up in the new economic model, young entrepreneurs have a more intuitive and profound understanding of the evolution of the social market economy. Compared with the traditional entrepreneurial groups, they are more likely to succeed in the new economic model. College students are the main force in the new era of China, and they need systematic and scientific entrepreneurship training. Therefore, multimedia technology and Internet technology are combined to improve the teaching quality of entrepreneurship education in colleges and universities, to change students’ learning styles, and to fully meet the students’ learning needs and the development needs of economic construction in the new era (Hou et al., 2019; Kurilova et al., 2019). However, there is little research on students’ innovation and entrepreneurship education, which is not conducive to the cultivation of students’ innovation and entrepreneurship capabilities. Therefore, college students should be educated by the teaching mode of the innovation and entrepreneurship education of young entrepreneurs based on information technology, and the influence of network entrepreneurship education and management system on students’ entrepreneurial literacy is analyzed.

Wei et al. (2019) studied the mediating effect of political skills and entrepreneurial opportunities on perceived entrepreneurship education and innovation and used structural equations to analyze the entrepreneurial information collected by the questionnaire. The results show that there is a positive correlation between entrepreneurship education and innovation, and political skills and entrepreneurial opportunities play a chain intermediary role between entrepreneurship education and innovation. Wu and Wu (2019) studied the impact of emotion on innovation behavior, and analyzed the mediating effect of job participation and behavior. The results show that positive emotion can mediate employees’ positive emotions, and engagement is the influence of positive emotion on innovation behavior. Boldureanu et al. (2020) studied the impact of entrepreneurial success cases on students’ entrepreneurial ability and attitudes and conducted experiments on students in qualitative and quantitative ways. The results show that after contacting successful entrepreneurial models, students can have higher social benefits for entrepreneurship. The study also finds that entrepreneurship education can change and arouse students’ entrepreneurial attitudes and enthusiasm by improving the teaching efficiency of developing entrepreneurial skills. Wu et al. (2019) studied the influence of a classroom response system on the interactive relationship between teachers and students and established an interactive classroom dominated by students. The results show that a classroom response system can promote the interaction between learners and learning content, arouse students’ learning enthusiasm and entrepreneurial motivation. Wu and Song (2019) studied the impact of social media on entrepreneurship curriculum from the perspective of learners. The results show that trust and profits are the important factors affecting the entrepreneurship curriculum. Obschonka et al. (2019) studied the impact of entrepreneurial thinking on organizational behavior, quantified the impact of personality traits on entrepreneurial passion in the organization, and tested this impact by using an alternative interpretation model. The results show that entrepreneurial personality traits can help inspire entrepreneurial enthusiasm. Zakieva et al. (2019) studied the impact of innovation activities on competitiveness and innovated an intellectual property law from the perspective of entrepreneurship education. The survey results show that 65% of teachers are interested in innovative entrepreneurship education courses, but they need to bear additional burdens and lack intellectual property rights in the field of innovation. According to the previous research, it is found that at this stage, an in-depth study is needed on the issue of entrepreneurship education in colleges and universities.

In order to improve the ability and quality of college students’ innovation and entrepreneurship, it is proposed that young entrepreneurs should use information technology to teach students how to start a business online and carry out situational simulation teaching through human-computer interaction (HCI). Since less importance is attached to entrepreneurship education and students’ entrepreneurial ability in colleges and universities, the current entrepreneurship education model should be optimized based on Internet technology. As the witness and participant of China’s economic transformation, young entrepreneurs have a good understanding of the entrepreneurial process under the new economic model. Based on HCI and psychology, the network entrepreneurial teaching mode of young entrepreneurs is analyzed, and the curriculum characteristics of different teaching modes are compared, and the influence of different entrepreneurial variables on students’ entrepreneurial psychological quality is discussed. The innovation of this study is to apply HCI to the network entrepreneurship teaching of young entrepreneurs, analyze the influence of optimized teaching mode and management system innovation on students’ entrepreneurial psychological quality, and judge the influence of network entrepreneurship teaching mode on students’ entrepreneurial ability and literacy. The research provides a reference for improving the teaching quality of entrepreneurship education in colleges and universities.

## Entrepreneurship Education and Students’ Entrepreneurial Psychology

### Entrepreneurship Education and Entrepreneurial Ability

The development of the entrepreneurship ecosystem in colleges and universities can effectively develop students’ entrepreneurial activities and promote the development of the social economy. In western countries, the entrepreneurship education ecosystem in colleges and universities is the main source of cultivating entrepreneurial talents for their own country, which promotes the development of the national economy. Therefore, it is necessary to establish a good environment for innovation and entrepreneurship education in colleges and universities to form a benign entrepreneurial ecosystem, so that the college students’ innovation and entrepreneurship can be improved. This system needs to include multiple subsystems, such as colleges and universities, students, families, husbands, and enterprises. Through the interaction between each system, a complete innovation and entrepreneurship education cultivation system can be established. As the backbone of the innovation and entrepreneurship education system, colleges and universities play an important role in the cultivation of entrepreneurial talents. As the receiver of innovation and entrepreneurship education, college students need to learn relevant innovation and entrepreneurship concepts and carry out entrepreneurial activities in practice.

The reason why entrepreneurship education courses are offered in Chinese universities is that the traditional university education system lacks the cultivation of college students’ innovation, entrepreneurial awareness, and entrepreneurial ability. As an important force to develop human society, innovation and entrepreneurship are important factors to enhance China’s international competitiveness (Fiore et al., 2019). Entrepreneurial ability refers to the ability of students to discover and create a new field, and to create new things in this field. The existing technologies and methods are developed and used to make new achievements. The cultivation of students’ entrepreneurial ability depends on financial, material, and manpower. Compared with employment ability, entrepreneurial ability pays more attention to the ability to find and solve problems.

The research contents of entrepreneurial psychology include the psychological characteristics of entrepreneurs, the behavior control of entrepreneurs, the communication between entrepreneurial teams, the conflict of entrepreneurial teams, and the humanized management of entrepreneurial teams. The cultivation and management of entrepreneurial mental health is an important part of entrepreneurial success. Therefore, in the process of implementing innovation and entrepreneurship teaching, colleges and universities do not require college students to leave school for entrepreneurship but cultivate their innovation and entrepreneurship spirit, enhance innovation and entrepreneurship awareness, and prevent possible risks in the process of entrepreneurship by learning innovation and entrepreneurship knowledge and skills. Therefore, as participants in the economic model of the new era, young entrepreneurs need to encourage and help college students with entrepreneurship and innovation ability, boldly invest in innovation and entrepreneurship activities, and provide spiritual guidance for entrepreneurial students (Komarova et al., 2019).

**FIGURE 1:**
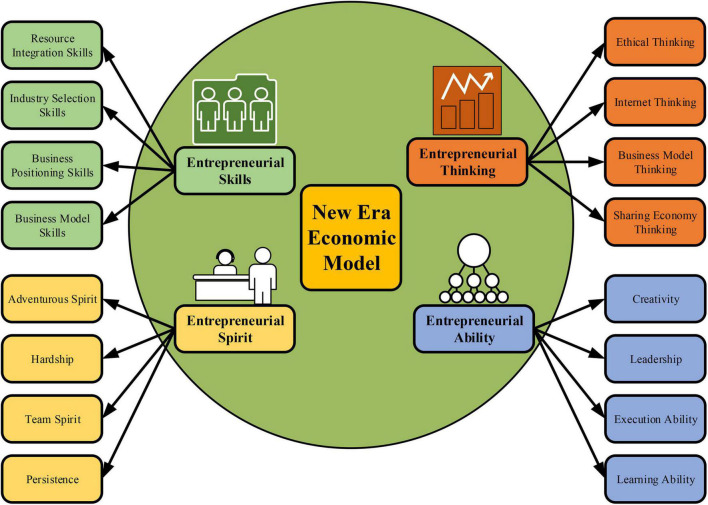
Entrepreneurial needs of college students under the new economic model.

At present, the current entrepreneurship education mode is relatively backward, with fixed time and place, a single teaching mode, and the teaching pattern of teachers’ lecturing basically and students’ learning passively. This cannot adapt to the development of mobile Internet and students’ entrepreneurial needs. And the teaching content and teaching objectives of colleges and universities are out of line with the actual needs. The content of the course lacks the characteristics of the times, compound, and applicability, and doesn’t present the innovation of knowledge structure brought by the development of science and technology (Linton and Klinton, 2019). The education system of colleges and universities advocates the key training of academic talents, while applied talents and entrepreneurial talents lack corresponding teaching modes, which cannot meet the needs of the development of vocational education and entrepreneurship education in the new era. Figure 1 is the requirements of students’ entrepreneurial ability under the new era economic model.

In terms of higher education, computer-aided teaching can effectively enhance the vividness and intuitiveness of entrepreneurship teaching, help teachers to simplify teaching procedures, and promote students’ in-depth understanding of the knowledge. Therefore, in the process of teaching practice, teachers need to apply information technology in the new era to conducting targeted teaching guidance to students and try to ensure the application of computer-aided teaching in entrepreneurship education (Aparicio et al., 2019; Tkachenko et al., 2019). Internet + education is the main development mode of higher education in the future. Therefore, an efficient innovation and entrepreneurship education system should consider the combination of innovation and entrepreneurship education and Internet technology. In network teaching, teachers, as the main body of education, need to use Internet thinking to reconstruct and exchange knowledge (García-Morales et al., 2020). And the teaching content also needs to connect with the Internet. The teaching method of entrepreneurship should take offline + online teaching mode so that students can make full use of their leisure time to learn the knowledge anytime.

### Analysis of the Teaching Mode of Entrepreneurship Education in Colleges and Universities Under the Innovation of Management System

Management system innovation is a key factor for the survival and development of an enterprise, and the factors affecting the success of different enterprises are not the same. With the comprehensive deepening reform of the social economy, young entrepreneurs are the main force in the process of the transformation of China’s new economic model, and they are creative, ideal, dare to question the *status quo*, and find opportunities. They are a group of young people who struggle to build an ideal world. They use business operations to analyze social problems and business rules to solve practical problems. Their purpose is not only to achieve commercial profits but also to organize and operate social welfare activities. In the globalization process, the competition for science and technology is becoming increasingly fierce. Young entrepreneurs should be more proactive in investing in the industry, joining the construction of the Belt and Road Initiative in China, and promoting the formation of an active “youth community,” bringing the spirit of dare to pursue their dreams and realize their values in the new era (Feng and Chen, 2020; Lv, 2020).

Innovation is the first driving force to guide the development of the social economy and the strategic support to realize the construction of China’s modern economic system. Entrepreneurship is an important force to promote the progress of human society and an important way to realize the industrialization and commercialization of innovation achievements. They are interdependent in the process of social and economic development. Therefore, when entrepreneurship education is carried out, it is advocated to link innovation and entrepreneurship, use innovation to drive entrepreneurship and entrepreneurship to drive innovation (Liang et al., 2021). Therefore, young entrepreneurs should be used as teachers of students’ entrepreneurship education. In the teaching process, teachers help students understand the development mode of entrepreneurship and solve problems by combining their own entrepreneurial experience and understanding the economic model of the new era, and enhance students’ entrepreneurial ability and entrepreneurial quality. Therefore, how to teach entrepreneurship courses in the teaching process is a problem that needs to be studied in depth. Common teaching methods include the traditional teaching method, case study method, and scene simulation method.

#### Traditional Narrative Method

Lecture teaching is a way of teaching students directly by teachers, which is widely used in college classroom teaching. With the advancement of the new curriculum teaching concept, lecture-based teaching is called inculcation teaching or cramming teaching, and independent, cooperative, and inquiry-based teaching methods are advocated in the new curriculum (Wang and Park, 2021). However, in lecturing teaching, teachers can transform complex and profound textbook knowledge into superficial or concrete knowledge that can be understood by students, and eliminate students’ mystery and confusion in learning entrepreneurial knowledge. And lecturing teaching can spread knowledge to students in the form of stereotypes, avoid the misunderstanding and misinterpretation of students caused by teachers in the dissemination of knowledge, and reduce the difficulties encountered by students in learning. Lecture teaching is a basic way, and the other teaching methods are based on it. Students can convert the knowledge taught into their ability through subtle or conscious learning (Wang, 2020; Deng et al., 2021).

However, there are also some shortcomings in the teaching method. When teachers use the teaching method, it will give students a false impression of being easy to learn. It seems that students only need to listen carefully to obtain the corresponding knowledge. However, in fact, students’ learning of knowledge needs to combine the knowledge they have mastered with independent thinking. In the process of imparting knowledge to teachers, they need to master the difficulties and questions encountered through independent thinking, and further use knowledge from one to another to promote the development of their abilities. The teaching method takes teachers as the center of teaching. Therefore, teachers’ understanding and thinking of knowledge affect students’ acceptance. In the teaching process, students will have psychological dependence, and they will wait for teachers to solve problems. In particular, the better the teacher speaks, the more serious the students’ psychological dependence is. This dependence will affect students’ active learning ability and independent thinking ability (Ghimire, 2020).

#### Case Study Method

The case study method originates from the research field of anthropology and sociology and is widely used in psychology and pedagogy. It refers to the continuous investigation of an individual or group for a long time, and the behavior development of the research object in the whole process of change, also known as the case analysis method (Li et al., 2019). In the case study, students need to collect, record and collate the case materials of one or more groups, and write the results of the study as a case report. In most cases, the research results of various discussion methods can be extended to the general situation, but some cases are used in practice after comparison. Therefore, the task of the case study is to organize the experience report for evaluating the process and to describe the case as the basis of the evaluation of general events. The case study method includes three types, namely, individual survey, group survey, and problem survey. The individual survey investigates one person as the research object. Group investigation is research on an organization or group. The problem survey is an investigation and study of a social problem or phenomenon (Hui et al., 2019).

#### Scene Simulation

The scene simulation method is under the guidance of classroom teaching, students stimulate a role or post in the actual scene through the simulation. And then in the scene environment created by teachers, their enthusiasm is aroused in skills training and training. In the understanding of the corresponding role, it is a typical interactive teaching mode. As an action-oriented mode, the scene simulation method is characterized by the content and scene that are close to the actual events, allowing students to actively participate in the role, and substituting and understanding the psychological state and behavior of the role (Corrales-Garay et al., 2020). Therefore, in the scene simulation teaching, the appropriate simulation teaching method can provide students with a more real entrepreneurial environment, enhance the interaction of teaching, train students’ language ability and problem-solving ability, understand the concept and mode of entrepreneurship in the new era, and establish a bridge between the theory and practice of entrepreneurship.

### Network Entrepreneurship Teaching Mode for Young Entrepreneurs Based on Human-Computer Interaction of Psychology

With the development of multimedia technology and computer performance, computer technology becomes an important means of teaching assistance and receives extensive attention. There are also great changes in the way of study. The establishment of a network teaching platform can greatly improve the learning efficiency of educational resources, improve user experience and enhance students’ interest in learning by establishing interactive teaching methods. After the scene simulation teaching method is stimulated, computer technology is applied to the teaching of young entrepreneurs’ entrepreneurship to arouse students’ learning interest. According to the relevant content of psychology, a HCI network education mode is designed based on user experience to improve the educational effect of the scene simulation teaching method.

**FIGURE 2:**
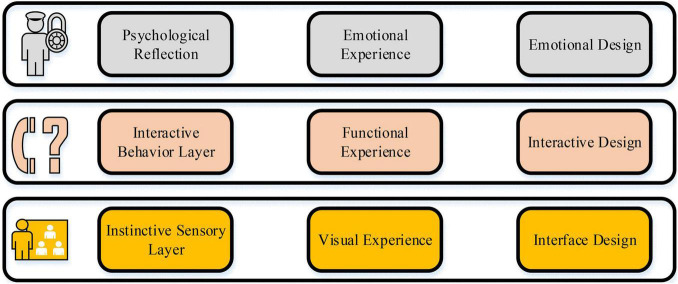
Educational mode based on users’ experience.

Attention is the direction and concentration of certain objects in psychological activities, and it is combined with human memory, consciousness, vision, and thinking. It belongs to common psychological characteristics. In most cases, students can only focus on one thing in the course of the class and filter out other external environmental stimuli, known as selective attention (Arquisola and Muanar, 2019). Students’ attention is easily attracted by moving objects, human faces, and other objects. Therefore, in the design of HCI, it is necessary to pay attention to these factors and study a network teaching system that is more in line with students’ cognitive habits. User experience is an important index to judge the performance of the HCI interface from the perspective of the user’s subjective feelings. Therefore, the establishment of a HCI teaching mode centered on students and teaching system can effectively improve students’ entrepreneurial teaching experience. The designed model mechanism is shown in Figure 2.

This mode is a comprehensive framework of user’ experience based on psychology and the results of students’ feelings and experience. This framework is a student-centered network teaching system (Zulfiqar et al., 2019; Zhang et al., 2020). The instinctive sensory layer represents the psychological instinct reaction caused by students’ feelings, consciousness, and performance. The factors that affect the instinct level include visual and auditory factors. In this process, students may have more intuitive inner feelings, resulting in the visual and browsing experience. The interactive behavior layer is the experience after HCI with the system. The influencing factors include operability, system performance, system function, teaching content, and information framework.

The content of the interactive behavior layer is based on the students’ learning process on the network teaching platform, which includes all the students’ behaviors on the network platform. The psychological reflection layer is the basis of the inner feelings of the instinct sensory layer and the interactive experience of the interactive behavior layer, which can bring users a more advanced and complex emotional experience. The design of the model should include pleasure, self-value, personality, and social significance. Therefore, the designed model is applied to the network entrepreneurship teaching of young entrepreneurs, and the influence of entrepreneurial education mode, entrepreneurial experience, and entrepreneurial ability on students’ entrepreneurial psychological quality is analyzed (Escolar-Llamazares et al., 2019).

### Survey Analysis

The influence of the network entrepreneurship teaching and management system on students’ entrepreneurial creativity, communication and leadership, and qualities is studied. The seniors in an economic and management university are selected as the subjects to conduct a questionnaire survey, and their majors are law, business, industry and commerce, finance, and logistics. The teaching methods of entrepreneurship education for the seniors are the traditional teaching method, case study method, and scene simulation method, and the students are investigated by a questionnaire at the end of the semester. The questionnaires are distributed and convenience sampling and random sampling are used for data statistics. 350 questionnaires are distributed, and 336 are recovered, with a recovery rate of 96.0%. The criterion for determining invalid questionnaires is whether the questionnaire is completed. The influence of the teaching mode and the management system innovation on students’ entrepreneurial ability, communication ability, and leadership is further explored.

The design of the questionnaire falls into two stages. The first is the measurement of the research variables, which include opportunity motivation, survival motivation, status awareness, and entrepreneurial ability. The Likert scale is used as the measurement index, and the subjective cognitive feelings are scored through the quantitative method; the second is the information statistics of college students’ entrepreneurship. The variables having been used in the domestic and foreign research are selected and combined with the relevant variables to improve the reliability of the questionnaire, SPSS 25.0 is used to analyze the data of the survey and the influencing factors of the entrepreneurship education, and the *t*-test (Student’s *t*-test) is used to make the small size of samples distribute normally (for example, *n* < 30). The difference can be inferred through the distribution theory and the average scores are compared to reveal whether the difference is significant.

## Discussion and Analysis of the Results of the Questionnaire Survey

### Reliability and Validity of the Questionnaire

To verify the reliability and validity of the designed questionnaire, Cronbach’s α coefficient, KMO (Kaiser-Meyer-Olkin), and Bartlett sphericity are used to test the reliability and validity of the questionnaire. The results are shown in Tables 1, 2.

**TABLE 1:** Reliability of questionnaire survey.

Research focuses	Cronbach’s Alpha	Number of items
Network entrepreneurship teaching	0.856	8
Innovation of management system	0.855	5
Students’ entrepreneurial ability	0.912	7

**TABLE 2:** Validity of questionnaire survey.

Research focuses	KMO	Approximate chi-square	Sig.
Network entrepreneurship teaching	0.833	252.466	0.000
Innovation of management system	0.853	148.964	0.000
Students’ entrepreneurial ability	0.865	867.647	0.000

Table 1 shows that the reliability of Cronbach’s α coefficient is above 0.85, and the α coefficient of each dimension, in general, is greater than the standard of 0.8, indicating that the scale data used in the explanatory text have high internal consistency and good stability. Table 2 indicates that the value of Bartlett sphericity is significant, and the KMO value is higher than 0.8 KMO. The other two are all above 0.85, indicating that the effect of the scale is good, and is suitable for factor analysis.

### *T*-test

In the questionnaire survey, the traditional teaching method, case study method, and scene simulation method are verified in analyzing their influence on students’ entrepreneurial learning. The results are shown in Table 3.

**TABLE 3:** Results of *t*-test.

Teaching methods		Traditional teaching method	Case study method	Scene simulation method
Traditional teaching method	Person correlation	1	0.595	0.584
Case study method	Person correlation	0.595	1	0.842
Scene simulation method	Person correlation	0.584	0.842	1

Table 3 shows that the traditional teaching method, case study method, and scene simulation method can improve students’ learning ability. The test result of the scene simulation method and the traditional teaching method is 0.584, the test result of the scene simulation method and the case study method is 0.842, and the test result of the case study method and the traditional teaching method is 0.595. This shows that scene simulation has a greater impact on students’ entrepreneurship education, and the results are better than other teaching methods in improving students’ entrepreneurial psychology and their entrepreneurial ability.

### Analysis of Factors Affecting Students’ Entrepreneurial Psychology

Multiple stepwise regression analysis is used to analyze the factors affecting students’ entrepreneurial psychology. The research variables included industries, survival motivation, opportunity motivation, and status cognition of young entrepreneurs so that the influence of other factors on the main effect can be avoided. The youth entrepreneurs involved in the survey come from the industries of education, foreign trade, industry, and software. The specific results of the correlation coefficient are shown in Table 4.

**TABLE 4:** Correlation coefficient.

Factors	Industries	Opportunity motivation	Survival motivation	Status cognition	Entrepreneurial ability
Industries	1				
Opportunity motivation	−0.041	1			
Survival motivation	0.067	0.492*	1		
Status cognition	0.128	0.576***	0.465***	1	
Entrepreneurial ability	0.117	0.347***	0.328**	0.238***	1

*, **, and ***, respectively, indicate P < 0.1, P < 0.05, and P < 0.01.

Table 4 shows that the significant correlation between survival motivation and opportunity motivation of young entrepreneurs and status cognition and entrepreneurial ability is 0.465, indicating that there is a significant correlation between young entrepreneurs’ motivation and entrepreneurial psychology. The entrepreneurial motivation of young entrepreneurs can positively affect entrepreneurial psychology, with a value of 0.041. The cognition of entrepreneurs’ professional status also significantly affects college students’ entrepreneurial psychology and attitudes, with a value of 0.576. In summary, the influencing factors of students’ entrepreneurial psychology include their motivation and status cognition, and status cognition has the greatest impact on entrepreneurial psychology. The study provides opportunity-driven students with positive entrepreneurial emotions. Compared with the results of the previous studies, the content of the study describes the influence of entrepreneurs’ occupational status cognition on students’ entrepreneurship more accurately and promotes the development of entrepreneurship education theory in colleges and universities.

## Conclusion

The study explores the entrepreneurial education in colleges and universities based on the optimization of HCI. In the research process, it is proposed to change the teaching method of college students’ entrepreneurship education, and the influence of entrepreneurship education and management network system on college students’ entrepreneurial psychology is studied. After analyzing the existing problems of traditional entrepreneurship education and the requirements of college students’ entrepreneurial ability under the new economic model, the study puts forward suggestions on using information technology to improve the teaching quality of college entrepreneurship education. Then it introduces different teaching methods for cultivating young entrepreneurs, and simulates entrepreneurship scenes through the network entrepreneurship teaching management system. Moreover, it analyzes the influence of different variables such as entrepreneurial teaching mode and entrepreneurial ability on students’ entrepreneurial psychology. The test result of the scenario simulation method and the traditional teaching method is 0.584, the test result of the scenario simulation method and the case learning method is 0.842, and the test result of the case learning method and the traditional teaching method is 0.595. This shows that the situational simulation method has a more obvious impact on students’ entrepreneurial psychology and entrepreneurial ability, and the cognition of occupational status affects students’ entrepreneurial psychology and entrepreneurial attitude.

The theoretical research contribution lies in analyzing the influence of entrepreneurial network teaching on students’ learning psychology; the practical enlightenment is to design a college entrepreneurship teaching system based on HCI and apply it to college entrepreneurship teaching classrooms. Compared with the research results of Lu (2021), the network entrepreneurship teaching and management system for young entrepreneurs based on HCI can have a positive impact on students’ entrepreneurial ability and entrepreneurial psychology, and help them acquire entrepreneurial knowledge and skills. This research provides a reference for exploring entrepreneurship education in colleges and universities. However, there are still some shortcomings. For example, the designed questionnaire analysis is not in-depth, and the sample size of the research is small. This problem will be optimized and resolved in the follow-up research.

## Data Availability Statement

The raw data supporting the conclusions of this article will be made available by the authors, without undue reservation.

## Ethics Statement

The studies involving human participants were reviewed and approved by Zhongnan University of Economics and Law Ethics Committee. The patients/participants provided their written informed consent to participate in this study. Written informed consent was obtained from the individual(s) for the publication of any potentially identifiable images or data included in this article.

## Author Contributions

ZW drafted and finalized the manuscript. RT collected and organized the data, researched and organized literature, and provided mentoring support. XC designed the framework of the manuscript and performed statistical analysis. All authors contributed to the article and approved the submitted version.

## Conflict of Interest

The authors declare that the research was conducted in the absence of any commercial or financial relationships that could be construed as a potential conflict of interest.

## Publisher’s Note

All claims expressed in this article are solely those of the authors and do not necessarily represent those of their affiliated organizations, or those of the publisher, the editors and the reviewers. Any product that may be evaluated in this article, or claim that may be made by its manufacturer, is not guaranteed or endorsed by the publisher.
